# Astragaloside IV and Saponins of Rhizoma Polygonati Cure Cyclophosphamide-Induced Myelosuppression in Lung Adenocarcinoma *via* Down-Regulating miR-142-3p

**DOI:** 10.3389/fonc.2021.630921

**Published:** 2021-04-20

**Authors:** Xian Gu, Ling-yu Zhu, Zhen-ye Xu, Ke-ping Shen

**Affiliations:** Department of Oncology, Longhua Hospital, Shanghai University of Traditional Chinese Medicine, Shanghai, China

**Keywords:** astragaioside IV, saponins of rhizoma polygonati, mir-142-3p, myelosuppression, HMGB1

## Abstract

Our previous study revealed that Shuanghuang Shengbai granule could cure the myelosuppression induced by cyclophosphamide (CTX) in lung cancer. However, its hematopoietic effects and molecular mechanisms remain not fully understood. Therefore, this study was intended to investigate the effects and the underlying mechanisms of Astragaloside IV (AS) and saponins of rhizoma polygonati (SRP), the two main bioactive ingredients of Shuanghuang Shengbai granule, on CTX-induced myelosuppression. CTX inhibited the proliferation and promoted apoptosis in bone marrow hematopoietic stem cells (BMHSCs), accompanied by the increased expression of miR-142-3p. AS and/or SRP treatment could alleviate CTX-induced cell injury and suppress the expression of miR-142-3p. Over-expression of miR-142-3p partially reversed the therapeutic effect of AS and/or SRP on CTX-induced cell injury in BMHSCs. Further mechanism exploration discovered that *HMGB1* was the target gene of miR-142-3p, and miR-142-3p negatively regulated the expression of HMGB1. To further explore the function of AS and/or SRP *in vivo*, we constructed a lung cancer xenograft combined with CTX-induced myelosuppression mouse model, and we found that AS and SRP remarkably reversed the CTX-induced reduction of white blood cells, bone marrow nucleated cells, and thymus index *in vivo* and did not affect the chemotherapy effect of lung cancer. Collectively, our results strongly suggested that AS and SRP could improve the hematopoietic function of myelosuppressed lung cancer mice, and their effects may be related to the inhibition of miR-142-3p expression in BMHSCs.

## Introduction

Lung cancer is the most common malignant tumor that causes the highest number of cancer-related death worldwide (1). Although significant progress has been made in the treatment strategies of lung cancer, the 5-year survival rate of lung cancer patients is still <20%, according to the latest epidemiological survey (2). Chemotherapy is the most effective systemic treatment for lung cancer (3). However, myelosuppression often occurs during chemotherapy for lung cancer patients (4). Chemotherapy-induced myelosuppression, which is often manifested as a decline in white blood cells (WBCs) and a series of hematopoietic dysfunction, is a main obstacle that restricts the progress of chemotherapy (5, 6). Therefore, it is important to improve the symptoms of myelosuppression for the insurance of the efficacy of chemotherapy. At present, drugs such as recombinant human granulocyte colony stimulating factor (G-CSF), erythropoietin (EPO), and thrombopoietin (TPO), which can protect bone marrow function and promote the recovery of hematopoietic function, are often added in the sequential therapy of conventional chemotherapy regimens (7, 8). EPO and TPO are expensive, while G-CSF has a potential safety hazard (potential role in promoting tumor cell proliferation) (9). Therefore, it is urgent to find inexpensive, safe, and effective drugs to ameliorate chemotherapy-induced myelosuppression.

Traditional Chinese medicine has increasingly been studied and used as a novel adjunctive treatment in different cancers (10). Shuanghuang Shengbai granule has been applied in cancer treatment for decades (11). According to our previous studies (12, 13), it had an ability to increase WBC recovery and alleviate myelosuppression during chemotherapy. Reportedly (13), WBCs were mainly differentiated from bone marrow hematopoietic stem cells (BMHSCs), and Shuanghuang Shengbai granule could promote the proliferation of CD34^+^SCA1^+^ BMHSCs *via* regulation of microRNAs. The results suggested that some bioactive ingredients of Shuanghuang Shengbai granule could inhibit BMHSC injury during chemotherapy, and screening these bioactive ingredients may provide a basis for the development of anti-myelosuppression drugs in the future.

Based on all these backgrounds and our previous studies, our present study focused on Astragaloside IV (AS) and saponins of rhizoma polygonati (SRP), the two main bioactive ingredients of Shuanghuang Shengbai granule, in bone marrow hematopoietic function and their underlying mechanisms and further exploring on whether these two bioactive ingredients could cure myelosuppression in a CTX-induced myelosuppression mouse model.

## Materials and Methods

### Animals and Isolation of BMHSCs

Male BALB/c nude mice (6-week-old, weighing 20 ± 2 g) were purchased from Vital River Laboratories (Jiaxing, Zhejiang, China) and maintained in the specific pathogen-free animal laboratory of Longhua Hospital (Shanghai, China) under controlled environmental conditions. All of the animal experiments and procedures were approved by the IACUC of Longhua Hospital. After being sacrificed, the femur and tibia of the mice were harvested. Then, bone marrow cells (BMCs) were flushed from the bones before adding 2 ml hemolysin (Sigma-Aldrich, St. Louis, MO, USA) to damage the red blood cells to form a single-cell suspension. The CD34^+^SCA1^+^ BMHSCs were isolated from BMCs by using a FACSCanto II flow cytometer (BD Biosciences, San Jose, CA, USA) according to the manufacturers' standard protocols (BioLegend, San Diego, CA, USA) as previously described (13). Data was analyzed by Flowjo software (Tree Star, San Diego, CA, USA).

### Cell Culture and miRNA Transfection

BMHSCs were cultured in StemSpan serum-free expansion medium (StemCell Technologies, Vancouver, BC, Canada) supplemented with 50 ng/ml stem cell factor (eBioscience, San Diego, CA, USA) and 50 ng/ml fibroblast growth factor-1 (PeproTech, Rocky Hill, NJ, USA) in a humidified atmosphere of 5% CO_2_ at 37°C. When the cells were in logarithmic phase, the cells were collected for transfection. The miR-142-3p mimics (142-3p) and corresponding negative control (NC) were synthesized and purchased from GenePharma Company (Shanghai, China). The full length of HMGB1 was subcloned into pcDNA3.1 vector to overexpress HMGB1 (HMGB1), and empty pcDNA3.1 as negative control (pcDNA) was purchased from GenePharma Company. Transfection was performed using Lipofectamine 3000 (Invitrogen, Carlsbad, CA, USA) according to the manufacturer's instructions.

### CCK-8 Assay

Cell viability was measured by using a Cell Counting-8 kit (CCK-8, Dojindo, Kumamoto, Japan). BMHSCs (5.0 × 10^3^/well) were seeded in 96-well microtiter plates (Corning Costar, NY, USA). After the indicated treatment, the CCK-8 solution (10 μl/well) was added into the wells and incubated for 2 h according to the manufacturer's protocol. Cell viability was reflected by measuring the absorbance of the converted dye at 450 nm with a microplate reader (Bio-rad, Hercules, CA, USA).

### BrdU Assay

Cell proliferation was measured by BrdU incorporation assay (Roche Applied Science, Germany). BMHSCs (5.0 × 10^3^/well) were plated in 96-well microtiter plates (Corning Costar). After the indicated treatment, the BrdU solution (10 μl/well) was added into the wells and incubated for 2 h. Then, the cells were fixed and denatured with FixDenat solution for 30 min incubated with anti-BrdU monoclonal antibody for 1.5 h and tetramethylbenzidine substrate for 15 min according to the manufacturer's protocol. Cell proliferation was reflected by measuring the absorbance of the converted dye at 450 nm with a microplate reader (Bio-rad).

### Annexin V-PI Staining Assay

Cell apoptosis was measured using Annexin V/PI staining assay as described previously (14). After the indicated treatment, BMHSCs were harvested and washed twice in PBS. Then, Annexin V-FITC and PI reagents were added into the cell suspension according to the manufacturer's instruction (KeyGen, Nanjing, Jiangsu, China). Each sample was quantitatively analyzed at Em = 488 nm and Ex = 570 nm by using a FACSCanto II flow cytometer (BD Biosciences), and then the fluorescence of each sample was analyzed by using the CellQuest software (Becton Dickinson, Franklin Lakes, NJ, USA).

### Luciferase Reporter Assay

The putative miR-142-3p binding site in the 3′UTR of HMGB1 was predicted by miRanda (http://www.microrna.org/microrna/home.do). The 3′UTR of HMGB1 containing the wild-type or mutant miR-142-3p binding site was constructed and ligated into pGL3 plasmid (Invitrogen) according to the manufacturer's instruction. Cells were co-transfected with plasmid pGL3- HMGB1-3′UTR-WT (WT)/pGL3-HMGB1-3′UTR-MUT (MUT) and miR-142-3p mimics (142-3p) or NC using Lipofectamine 3000. At 48 h later, cells were collected, and relative luciferase activities were detected by a dual-luciferase reporter assay system (Promega, Madison, WI, USA) according to the manufacturer's instruction. Renilla luciferase was used for normalization.

### Real-Time Quantitative PCR

RNA was extracted from cells using Trizol Reagent (Invitrogen) and reversed transcriptase into cDNA by using a Prime Script RT reagent kit (TaKaRa, Dalian, China). Then, real-time quantitative PCR (RT-qPCR) was performed in triplicate using SYBR Premix Ex Taq Kit (Takara) according to the manufacturer's instruction. U6 and GAPDH were used as internal controls for normalizing and quantifying the expression of miR-142-3p and HMGB1, respectively. The relative expression levels of miR-142-3p and HMGB1 were calculated by using the 2^−ΔΔCt^ method. The primer sequences for miR-142-3p were 5′-GTCGTATCCAGTGCAGGG-3′ (forward) and 5′-CGACGTGTAGTGTTTCCAT-3′ (reverse). The primer sequences for HMGB1 mRMA were 5′-GATGGGCAAAGGAGATCCTA-3′ (forward) and 5′-CTTGGTCTCCCTTTGGGG-3′ (reverse).

### Western Blot Analysis

Proteins were extracted by RIPA buffer (Sigma-Aldrich) containing 1% protease inhibitor (cocktail, CalbioChem, Darmstadt, Germany), and the concentration of proteins was determined by using a BCA Protein Assay Kit (Beyotime, Shanghai, China). Equal amounts of proteins (70 μg proteins per sample) were denatured at 95°C for 5 min and then separated by SDS-PAGE system (Bio-rad). After transferring the electrophoretic protein onto PVDF membranes (Millipore, Billerica, MA, USA) by electrotransfer (Bio-rad) and blocking with 5% non-fat milk at room temperature for 90 min, the proteins were probed with primary antibodies against Bcl-2 (ab196495), Bax (ab216494), cleaved caspase3 (c-cas3, ab214430), caspase3 (cas3, ab13847), HMGB1 (ab18256), and GAPDH (ab181602) (Abcam, Cambridge, MA, USA) at 4°C overnight, followed by incubation with the appropriate peroxidase-conjugated secondary antibodies at room temperature for 1 h. Blots were developed by chemiluminescence (Beyotime), and then an X-ray film was scanned. The gray ratios of the target protein bands were accurately determined by an Image-J analysis system (NIH, Rockville Pike, Bethesda, MD, USA).

### Animal Model

Lung cancer xenograft model was constructed with A549 cell inoculation according to our previous research (13). When the tumor volume was about 200 mm^3^ [measured with a vernier caliper, the tumor volume was calculated as described previously (13)], then mice with similar tumor size were selected and randomly divided into control group (Ctrl) (*n* = 10 mice), CTX group (model) (*n* = 10 mice), CTX + AS group (*n* = 10 mice), CTX + SRP group (*n* = 10 mice), and CTX + AS + SRP group (*n* = 5 mice). CTX (Jiangsu Hengrui Medicine Co. Ltd, Jiangsu, China) was used to establish a myelosuppression mouse model. AS and SRP were obtained from the drug preparation center of Longhua Hospital. The mice in the CTX model group were given CTX (30 mg/kg/d) intraperitoneally (i.p.) for 14 days, and the control group received an equal volume of saline. The treatment groups were i.p. injected with AS (20 mg/kg/day), SRP (40 mg/kg/day), or AS (20 mg/kg/day) + SRP (40 mg/kg/day) for 14 days at 24 h after CTX administration. The mice were sacrificed, and their BMCs were collected for further analysis.

### Determination of Bone Marrow Hematopoietic Function

We investigated the effects of AS and SRP on bone marrow hematopoietic function, including peripheral blood, bone marrow nucleated cells (BMNCs) count, and thymus/spleen indexes in mice.

For general blood indexes detection, all mice's peripheral blood was collected from the eye socket vein. Then, WBCs, red blood cells (RBCs), and platelets (PLTs) were counted with an automatic blood cell analyzer (DxH 800TM, Beckman Coulter, Miami, FL, USA).

For BMNCs count, RBCs in BMC suspension were damaged with hemolysin and then centrifuged for 15 min at 2,000 rpm. The BMNCs were removed and washed with sterile PBS and then suspended and counted with an inverted optical microscope (CX43, Olympus, Tokyo, Japan).

For determination of thymus/spleen indexes, the mice were sacrificed, and then the thymus and spleen were obtained and weighed: thymus index = thymus weight/body weight; spleen index = spleen weight/body weight.

### Statistical Analysis

Data were expressed as means ± SD (standard deviation). The data were assessed using one-way ANOVA (*post hoc* test: LSD). Statistical analysis was performed by GraphPad Prism software (La Jolla, CA, USA). *P* < 0.05 was considered a statistically significant difference.

## Results

### AS and SRP Alleviated CTX-Induced Cell Injury in BMHSCs

**Figure 1:**
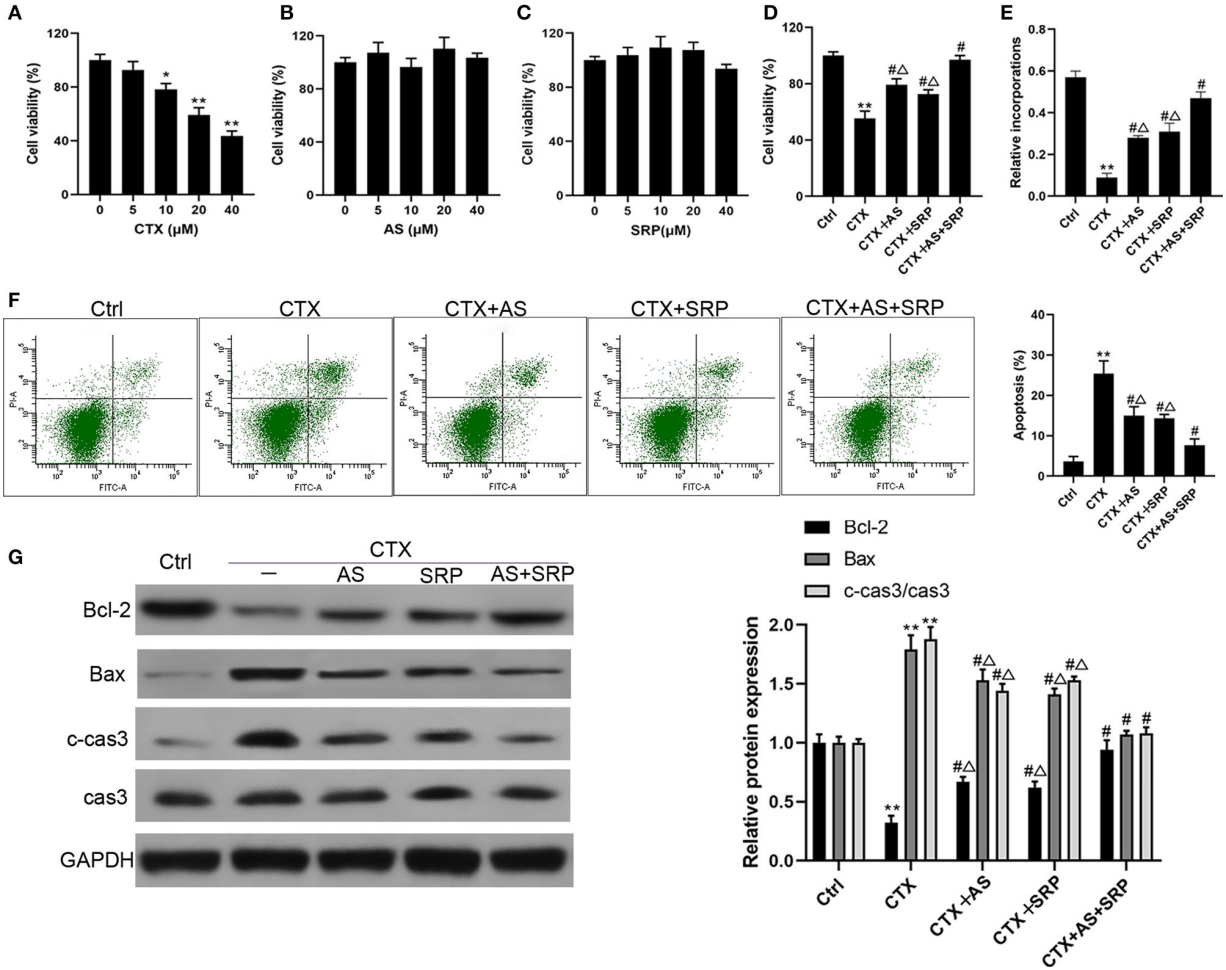
Astragaloside IV (AS) and saponins of rhizoma polygonati (SRP) alleviated cyclophosphamide (CTX)-induced cell injury in bone marrow hematopoietic stem cells (BMHSCs). BMHSCs were treated with CTX, AS, or SRP at various concentrations that range from 0 to 40 μM, 20 μM CTX + 5 μM AS, or 20 μM CTX + 10 μM SRP for 48 h. **(A**–**D)** Cell viability was measured by CCK-8 assay. **(E)** Cell proliferation was measured by BrdU assay. **(F)** Apoptosis was detected by Annexin V-PI staining. **(G)** Protein expressions of Bcl-2, Bax, and c-cas3/cas3 were determined by Western blot. ^*^P < 0.05, ^**^P < 0.01 vs. Ctrl group; ^#^*P* < 0.01 vs. CTX-alone group; ^Δ^*P* < 0.05 vs. CTX + AS + SRP group; *n* = 6.

At first, we examined the effect of CTX, AS, or SRP treatment alone on cell viability in BMHSCs. The results (Figures 1A–C) showed that, after treatment with CTX, AS, or SRP at a concentration range from 0 to 40 μM for 48 h, CTX inhibited cell viability in a concentration-dependent manner, while AS or SRP treatment alone had no harmful effect on it in BMHSCs. We next investigated whether AS and SRP could alleviate CTX-induced cell injury in BMHSCs. As shown in Figures 1D–F, AS and SRP could inhibit CTX-induced cell viability reduction, proliferation attenuation, and apoptosis. In addition, AS combined with SRP had an obvious synergistic protective effect in BMHSCs. Moreover, AS and SRP did not affect the killing effect of CTX in A549 cells (Supplementary Figure 1). We further investigated the effect of AS and SRP on the expressions of CTX-induced apoptosis-related proteins. The Western blot results illustrated that AS and SRP triggered a rise of Bcl-2 and a decline of Bax and cleaved caspase-3 under CTX condition (Figure 1G). All the data suggested that AS and SRP may be the potential anti-myelosuppression drugs during chemotherapy.

### AS and SRP Prevented CTX-Induced Cell Injury Through Down-Regulation of miR-142-3p in BMHSCs

**Figure 2:**
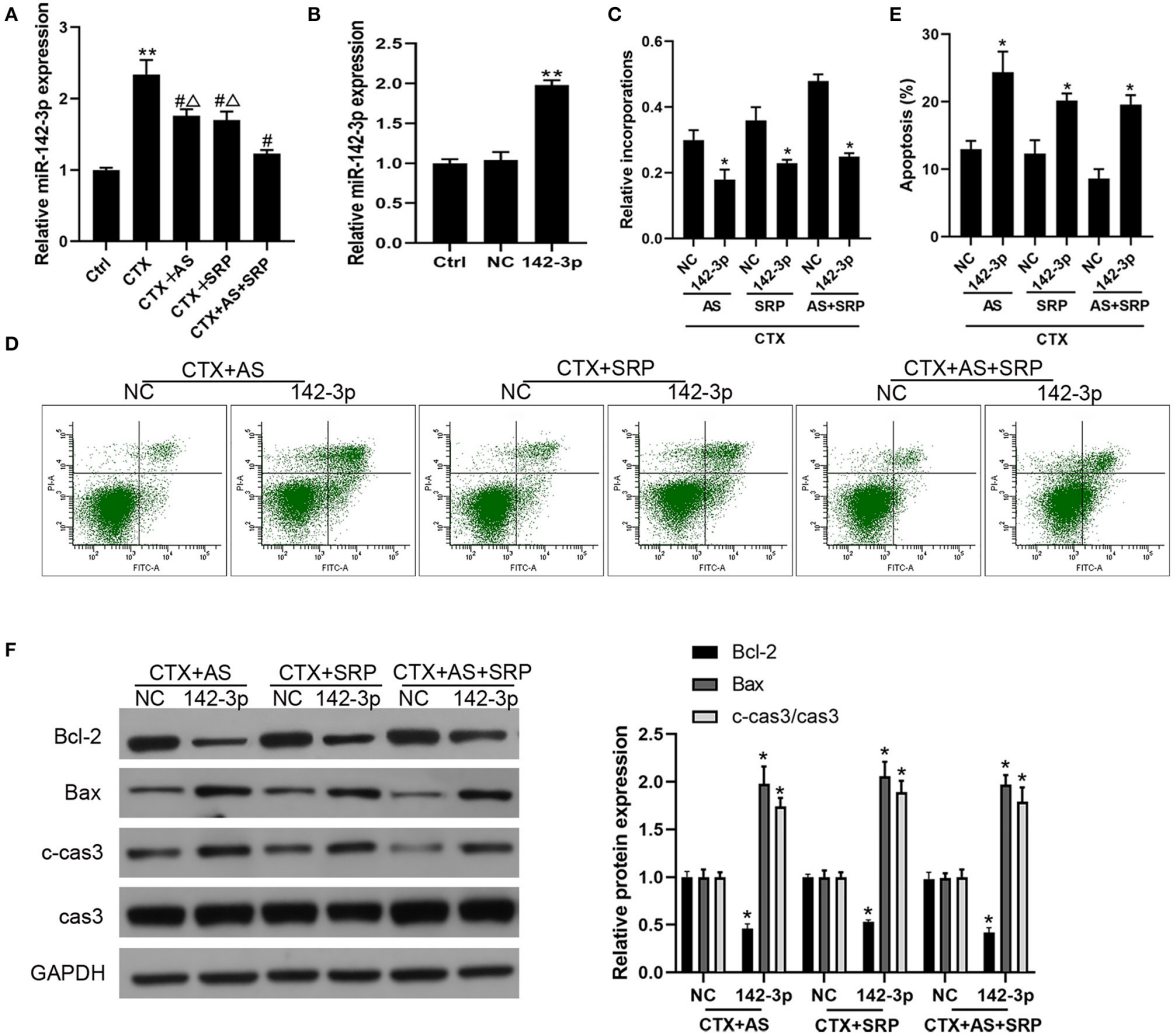
Over-expression of miR-142-3p abolished the inhibitory effect of Astragaloside IV (AS) and saponins of rhizoma polygonati (SRP) on CTX-induced cell injury in bone marrow hematopoietic stem cells (BMHSCs). **(A)** BMHSCs were treated with 20 μM CTX, 20 μM CTX + 5 μM AS, 20 μM CTX + 10 μM SRP, or 20 μM CTX + 5 μM AS + 10 μM SRP for 48 h. The expression of miR-142-3p was detected by RT-qPCR. **(B)** After transfection with miR-NC or miR-142-3p mimic, the expression of miR-142-3p was detected by RT-qPCR. **(C)** Cell proliferation was measured by BrdU assay. **(D,E)** Cell apoptosis was detected by Annexin V-PI staining. **(E)** Protein expressions of Bcl-2, Bax, and c-cas3/cas3 were determined by Western blot. **P < 0.01 vs. Ctrl group; *P < 0.05 vs. NC group; ^#^*P* < 0.01 vs. CTX alone group; ^Δ^*P* < 0.05 vs. CTX + AS + SRP group; *n* = 6.

Our previous study indicated that Shuanghuang Shengbai granule could reverse the up-regulation of miR-142-3p induced by CTX (13). We hypothesized that AS and SRP could interact with miR-142-3p in BMHSCs, and the RT-qPCR results disclosed that CTX augmented miR-142-3p expression in BMHSCs, which was consistent with our previous findings, while both AS and SRP partially reversed the effects of CTX on miR-142-3p expression (Figure 2A). Hence, we speculated that miR-142-3p expression may be involved in AS and SRP preventing CTX-induced cell injury in BMHSCs. To investigate the potential interaction among miR-142-3p expression, AS, SRP, and CTX-induced cell injury in BMHSCs, we examined the role of miR-142-3p over-expression in it. As shown in Figure 2B, miR-142-3p level was enhanced after transfection of miR-142-3p mimic. In the CTX-induced cell injury model, over-expression of miR-142-3p could reverse the protective effect of AS and SRP on BMHSCs by inhibiting cell proliferation (Figure 2C) and promoting apoptosis (Figures 2D,E). The phenomenon was also verified by Western blot. Compared to CTX + AS + NC group, CTX + SRP + NC group, or CTX + AS + SRP + NC group, over-expression of miR-142-3p induced the drop of Bcl-2 protein level and the rise of Bax and caspase-3 protein level (Figure 2F).

### MiR-142-3p Directly Targeted HMGB1 in BMHSCs

**Figure 3:**
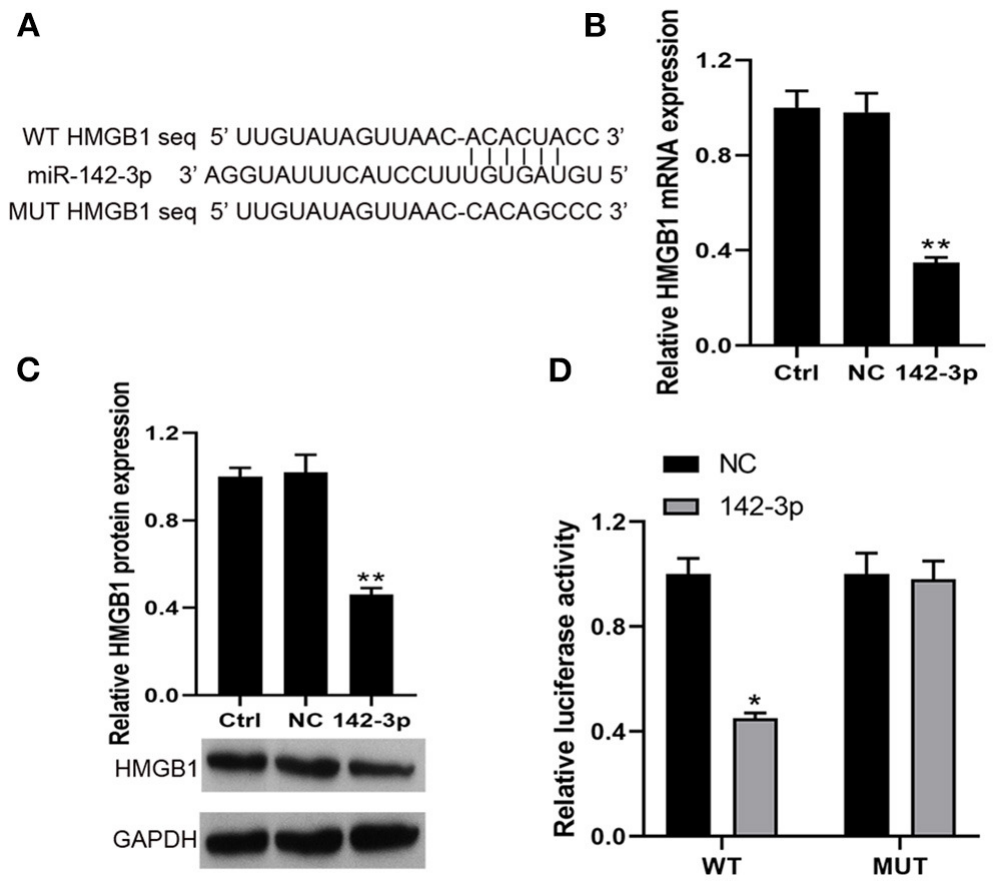
HMGB1 was a direct target of miR-142-3p. **(A)** The potential miR-142-3p binding sequence of HMGB1 3′UTR and the mutant sequence of HMGB1. **(B,C)** After transfection with miR-NC or miR-142-3p mimic, HMGB1 mRNA and protein levels were determined by RT-qPCR and Western blot in bone marrow hematopoietic stem cells, respectively. **(D)** The analysis of the relative luciferase activities of HMGB1. **P < 0.01 vs. Ctrl group; *P < 0.05 vs. NC group; *n* = 6.

Based on miRanda, we identified a putative miR-142-3p binding site in the 3′UTR of HMGB1 (Figure 3A). Then, our RT-qPCR and Western blot results found that miR-142-3p transfection could significantly decrease HMGB1 mRNA and protein expression in BMHSCs (Figures 3B,C). Moreover, dual-luciferase reporter assays also showed that co-transfection of miR-142-3p with the wild-type 3′UTR led to a 55% loss of luciferase reporter activity (Figure 3D); however, mutation of the miR-142-3p binding site in the HMGB1 3′UTR abolished the effect of miR-142-3p (Figure 3D), which indicated that miR-142-3p negatively regulates HMGB1 in BMHSCs by directly targeting the 3′UTR of HMGB1.

### Over-expression of HMGB1 Reversed the Functional Roles of miR-142-3p

**Figure 4:**
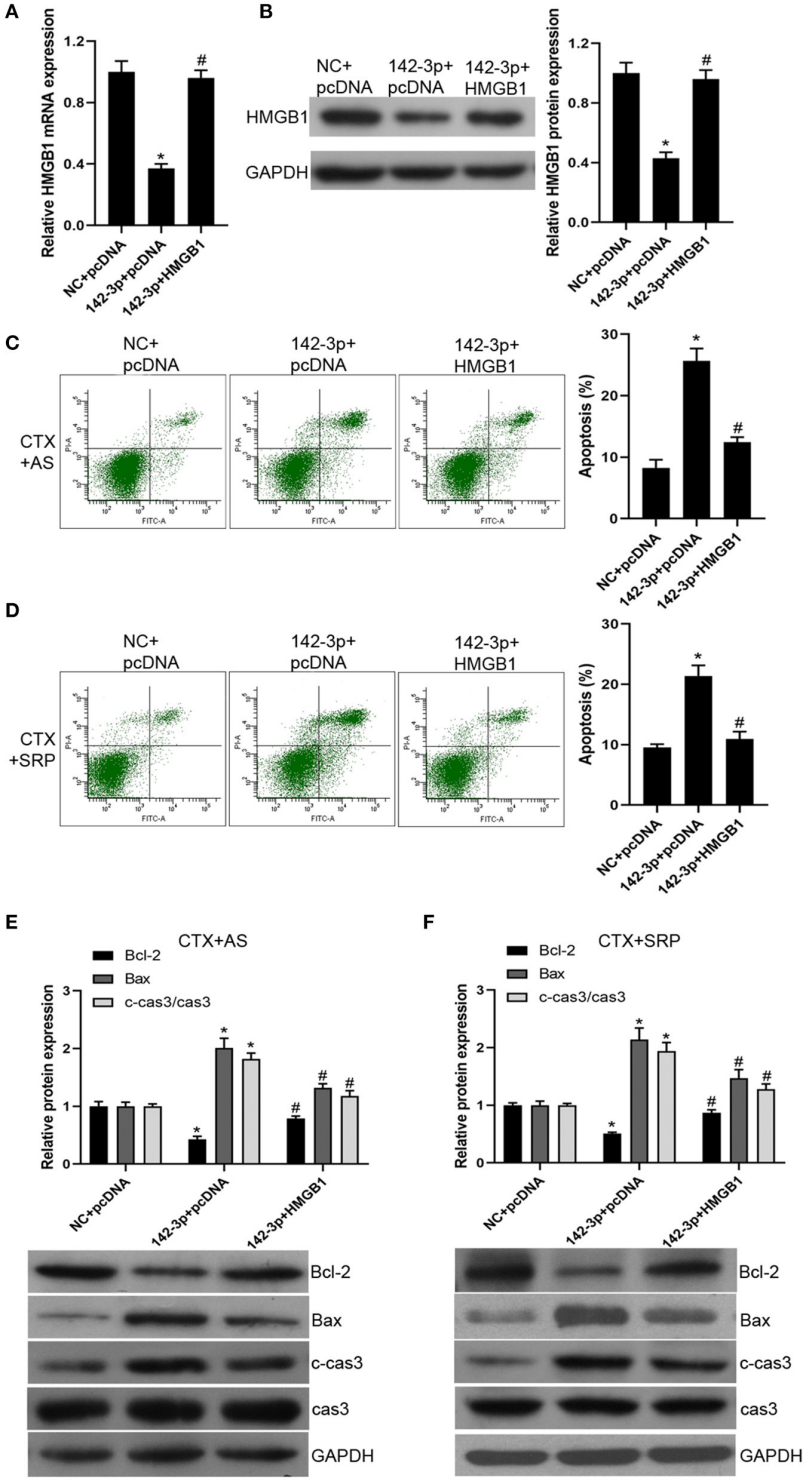
Over-expression of HMGB1 reversed the effect of miR-142-3p on apoptosis in bone marrow hematopoietic stem cells. **(A,B)** After co-transfection with miR-142-3p mimic and pcDNA-HMGB1, HMGB1 mRNA and protein levels were determined by RT-qPCR and Western blot, respectively. **(C,D)** Cell apoptosis was detected by Annexin V-PI staining under CTX + AS **(C)** or CTX + SRP **(D)** condition. **(E,F)** Protein expressions of Bcl-2, Bax, and c-cas3/cas3 were determined by Western blot. Cell apoptosis was detected by Annexin V-PI staining under CTX + AS **(E)** or CTX + SRP **(F)** condition. *P < 0.05 vs. NC + pcDNA group; ^#^*P* < 0.05 vs. 142-3p + pcDNA group; *n* = 6.

To further confirm that over-expression of miR-142-3p inhibited the therapeutic effect of AS and SRP on CTX-induced cell injury in BMHSCs through a HMGB1-dependent mechanism, we conducted functional rescue assay. Our data showed that, compared with miR-142-3p mimics + pcDNA3.1 vector group, the expression of HMGB1 was dramatically increased after co-transfection of miR-142-3p mimics and pcDNA-HMGB1 vector in BMHSCs (Figures 4A,B). The Annexin V-PI staining results showed that the promotion effect of miR-142-3p on apoptosis was reversed by pcDNA-HMGB1 vector transfection in BMHSCs under CTX + AS or CTX + SRP condition (Figures 4C,D). At the same time, the expressions of Bax and cleaved caspase3 were decreased, and the expression of Bcl-2 was increased in miR-142-3p-overexpressed BMHSCs after exogenous up-regulation of HMGB1 under CTX + AS or CTX + SRP condition (Figures 4E,F). Therefore, our data clearly confirmed that over-expression of miR-142-3p promoted apoptosis in BMHSCs by directly targeting HMGB1 under CTX + AS or CTX + SRP condition.

### AS and SRP Cure Myelosuppression *in vivo*

**Figure 5:**
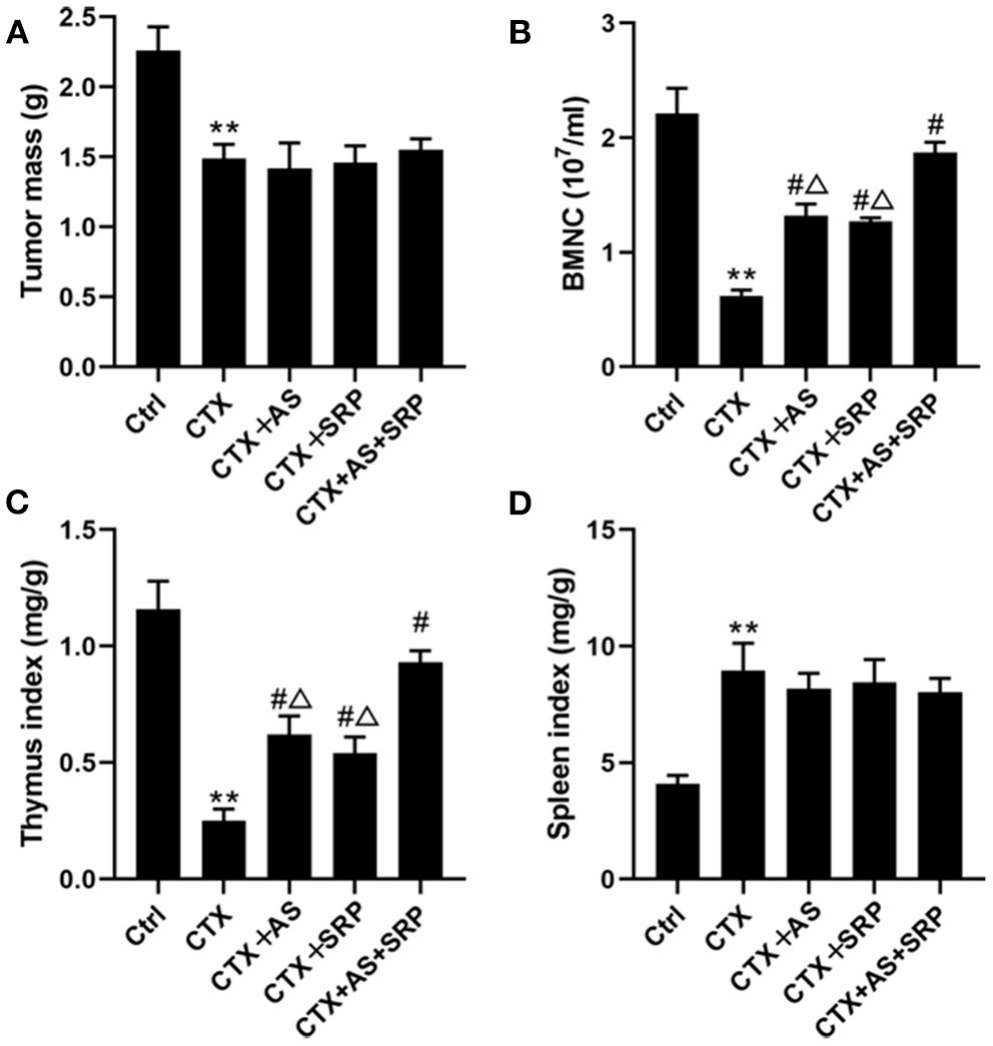
Astragaloside IV (AS) and saponins of rhizoma polygonati (SRP) cures myelosuppression *in vivo*. Effect of AS and SRP on tumor mass **(A)**, bone marrow hematopoietic stem cell counts **(B)**, thymus index **(C)**, and spleen index **(D)** in lung cancer xenograft combined with cyclophosphamide (CTX)-induced myelosuppression in mice. **P < 0.01 vs. Ctrl group; ^#^*P* < 0.05 vs. CTX-alone group; ^Δ^*P* < 0.05 vs. CTX + AS + SRP group; *n* = 10 or 5.

To further explore the function of AS and/or SRP *in vivo*, we constructed a lung cancer xenograft combined with a CTX-induced myelosuppression mouse model. Although AS and/or SRP did not affect the efficacy of CTX chemotherapy (compared with CTX-alone group, the tumor mass in CTX + AS, CTX + SRP, and CTX + AS + SRP groups was not changed significantly) (Figure 5A), they could alleviate the reduction of WBCs (Table 1) and BMNCs (Figure 5B) caused by CTX chemotherapy. In addition, we examined the thymus/spleen indexes in all the groups, and the data showed that, compared with the control group, the thymus index was decreased, and the thymus index was increased in CTX groups. Compared with the CTX-alone group, both AS and SRP remarkably increased the thymus index, while their effects on spleen index were not significant (Figures 5C,D). We also examined the expressions of miR-142-3p and HMGB1 in BMHSCs isolated from the mice in these groups, and the RT-qPCR and Western blot results showed that, compared with the CTX-alone group, the expression of miR-142-3p was significantly decreased, and the mRNA and protein expression of HMGB1 were significantly increased in the CTX + AS, CTX + SRP, and CTX + AS + SRP groups (Supplementary Figure 2). All the data suggested that AS and/or SRP could cure myelosuppression after CTX chemotherapy, and the effects may be related to their actions in regulating miR-142-3p and HMGB1 expressions in BMHSCs.

**Table 1:** Effect of Astragaloside IV and saponins of rhizoma polygonati on general blood indexes in mice.

**Groups**	**White blood cells (× 10^**9**^/L)**	**Red blood cells (× 10^**12**^/L)**	**Platelets (× 10^**9**^/L)**
Ctrl	8.73 ± 0.59	7.52 ± 0.29	124.35 ± 6.42
CTX	3.04 ± 0.21^**^	7.56 ± 0.77	116.74 ± 4.53
CTX + AS	8.36 ± 0.75^#^	7.98 ± 0.52	107.56 ± 5.17
CTX + SRP	7.17 ± 0.65^#^	7.69 ± 0.67	110.38 ± 4.21
CTX + AS + SRP	8.42 ± 0.46^#^	7.84 ± 0.33	114.15 ± 5.62

** *P < 0.01 vs. Ctrl group*;

# *P < 0.01 vs. CTX-alone group; n = 6*.

## Discussion

At present, chemotherapy is still a relatively effective and widely used treatment strategy for malignant tumors. However, due to the serious multi-system toxic and side effects of chemotherapy drugs, it may lead to the decline of immune function and bone marrow hematopoietic dysfunction, thus leading to the interruption of chemotherapy (5, 6). The currently used anti-myelosuppression drugs are expensive and do not work as well as expected (7–9), so it is very necessary to find inexpensive, safe, and effective anti-myelosuppression drugs.

CTX is a broad-spectrum anti-tumor drug that has inhibitory effects on a variety of tumors including lung cancer (15, 16). The most common side effect of CTX is myelosuppression (16). WBC reduction of peripheral blood is the main clinical manifestation of chemotherapy-induced myelosuppression (13, 17). The main source of WBCs is differentiation of BMHSCs (13). Therefore, BMHSC injury can cause myelosuppression. According to our previous studies (12, 13), we investigated the effect of AS and/or SRP on CTX-induced myelosuppression *in vitro*, and our findings showed that they could alleviate CTX-induced cell injury in BMHSCs. As a consequence of the number of BMNCs, a direct indicator of bone marrow hematopoietic function, high BMNC content generally represents strong hematopoietic function (18). Thymus index is one of the important indexes of cellular immunity, and it is closely related to hematopoietic function after chemotherapy (19). Therefore, we further examined these indicators to evaluate the anti-myelosuppression and hematopoietic effects of AS and/or SRP in lung cancer xenograft combined with CTX-induced myelosuppression mice. As a result, AS and/or SRP reversed the CTX-induced reduction of WBCs, BMNCs, and thymus index *in vivo* and, at the same time, did not affect the efficacy of CTX chemotherapy *in vitro* and *in vivo*. Collectively, these results expounded that AS and SRP could improve myelosuppression and hematopoietic function, and they may be the prospective anti-myelosuppression drugs during chemotherapy.

We next investigated why could AS and/or SRP inhibit CTX-induced myelosuppression. It is well-known that the increased expression of pro-apoptotic factor Bax and the decreased expression of anti-apoptotic factor Bcl-2 can activate the cleavage of casepase3 and induce apoptosis (20). In the present study, CTX induced BHMSC apoptosis by down-regulation of Bcl-2 and up-regulation of Bax, with activation of caspase3. This result was consistent with the fact that CTX causes myelosuppression (21). We observed that AS and/or SRP could partially reverse the effect of CTX on the expression of the molecules mentioned above, suggesting that As and SRP inhibited CTX-induced BMHSC injury by triggering the increase of Bcl-2 and the decrease of Bax and cleaved caspase-3.

We further investigated how AS and/or SRP affects the expressions of apoptosis-related proteins and prevent CTX-induced cell injury in BMHSCs. Based on our previous results (13), we hypothesized that miR-142-3p expression may be involved in this study. As we have predicted, both AS and SRP reversed the CTX-induced up-regulation of miR-142-3p expression. There was an evidence that miR-142-3p accelerated the formation and differentiation of hematopoietic stem cells (22). To further confirm its function, miR-142-3p mimic was used to simulate endogenous miR-142-3p expression, and the results showed that miR-142-3p up-regulation counteracted the inhibitory effect of AS and/or SRP on CTX-induced apoptosis of BMHSCs. It hinted that AS and SRP alleviated CTX-induced cell injury in BMHSC *via* down-regulation of miR-142-3p.

MiRNAs play biological roles by negatively regulating target genes (23). Through the target gene prediction software, we found that miR-142-3p and the HMGB1 3′-UTR region had the complementary base pairing binding sequence. HMGB1 has been shown to be involved in regulating cell survival and death mainly through DNA repair pathways (24–26). Xiao et al. reported that miR-142-3p inhibited cell proliferation and induced cell apoptosis in non-small cell lung cancer cells by targeting HMGB1 (27). We further confirmed that miR-142-3p directly targeted HMGB1 by RT-qPCR, Western blot, and luciferase reporter assay. Importantly, we also showed that the miR-142-3p overexpression-induced apoptosis in BMHSCs was partly reversed by up-regulating HMGB1 expression under CTX + AS or CTX + SRP condition.

Collectively, our data demonstrated that AS and SRP alleviated CTX-induced cell injury in BMHSCs *via* up-regulation of HMGB1 through inhibition of miR-142-3p, improved the hematopoietic function, and cured myelosuppression in CTX-induced myelosuppression mice.

## Data Availability Statement

The raw data supporting the conclusions of this article will be made available by the authors, without undue reservation.

## Ethics Statement

The animal study was reviewed and approved by IACUC of Longhua Hospital.

## Author Contributions

XG performed the experiments and drafted the manuscript. Z-yX and K-pS participated in the design of this study. L-yZ performed the statistical analysis. K-pS participated in scientific discussion of the data and reviewed the manuscript.

## Conflict of Interest

The authors declare that the research was conducted in the absence of any commercial or financial relationships that could be construed as a potential conflict of interest.
