# Supplementary material for: Astragaloside IV and Saponins of Rhizoma Polygonati Cure Cyclophosphamide-Induced Myelosuppression in Lung Adenocarcinoma via Down-Regulating miR-142-3p
Source: Front Oncol. 2021 Apr 20;11:630921. doi: 10.3389/fonc.2021.630921 (PMC8093395; doi:10.3389/fonc.2021.630921)
Supplement: Supplementary file 1 [file Data_Sheet_1.doc]

*1.1 Cell culture, treatment and CCK-8 assay*

A549 cells were purchased from ATCC (Manassas, VA, USA) and cultured in DMEM medium (Gibco, Grand Island, NY, USA) containing 10% fetal bovine serum (FBS) (Gibco), 100 mg/ml penicillin and 100 mg/ml streptomycin in a humidified atmosphere of 5% CO2 at 37℃. When the cells were in logarithmic phase, the cells were harvested and then re-inoculated into 96-well plate (5000 cells/well). The cells were treated with 20 μM CTX, 5 μM AS, 10 μM SRP, 5 μM AS+10 μM SRP, 20 μM CTX+5 μM AS, or 20 μM CTX+10 μM SRP for 48 h, then cell viability was measured by CCK-8 assay. The results showed that AS and/or SRP treatment did not affect cell viability in baseline (without CTX) (Supplemental Figure 1A) and also did not affect the killing effect of CTX in A549 cells (Supplemental Figure 1B).

**
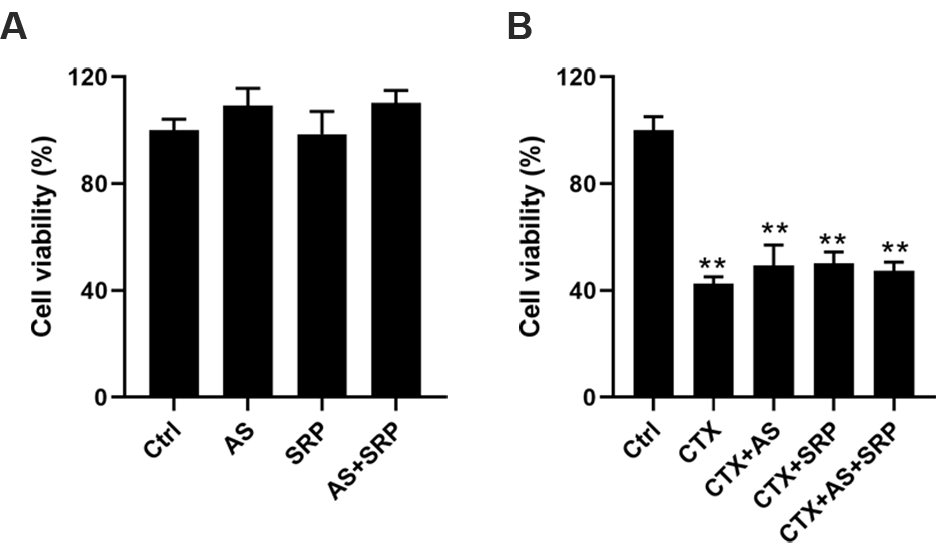
**

**Supplemental Figure 1. Effect of AS and/or SRP treatment on cell viability. (A)** Cell viability of A549 in baseline (without CTX). **(B)** Cell viability of A549 under CTX condition. Data were expressed as means±SD, ***P*<0.01 *vs.* Ctrl group; *n*=3.

*1.2 Isolation of BMHSCs from the mice in various groups, RT-qPCR and Western blot*

BMCs were collected from the mice in various groups. And, the CD34^+^SCA1^+^ BMHSCs were isolated from BMCs by flow cytometry. The expression of miR-142-3p and HMGB1 mRNA expression levels were detected by RT-qPCR, and the protein expression level was detected by Western blot. The results showed that, compared with CTX alone group, the expression of miR-142-3p was significantly decreased and the mRNA and protein expression of HMGB1 was significantly increased in CTX+AS, CTX+SRP and CTX+AS+SRP groups (Supplemental Figure 2).


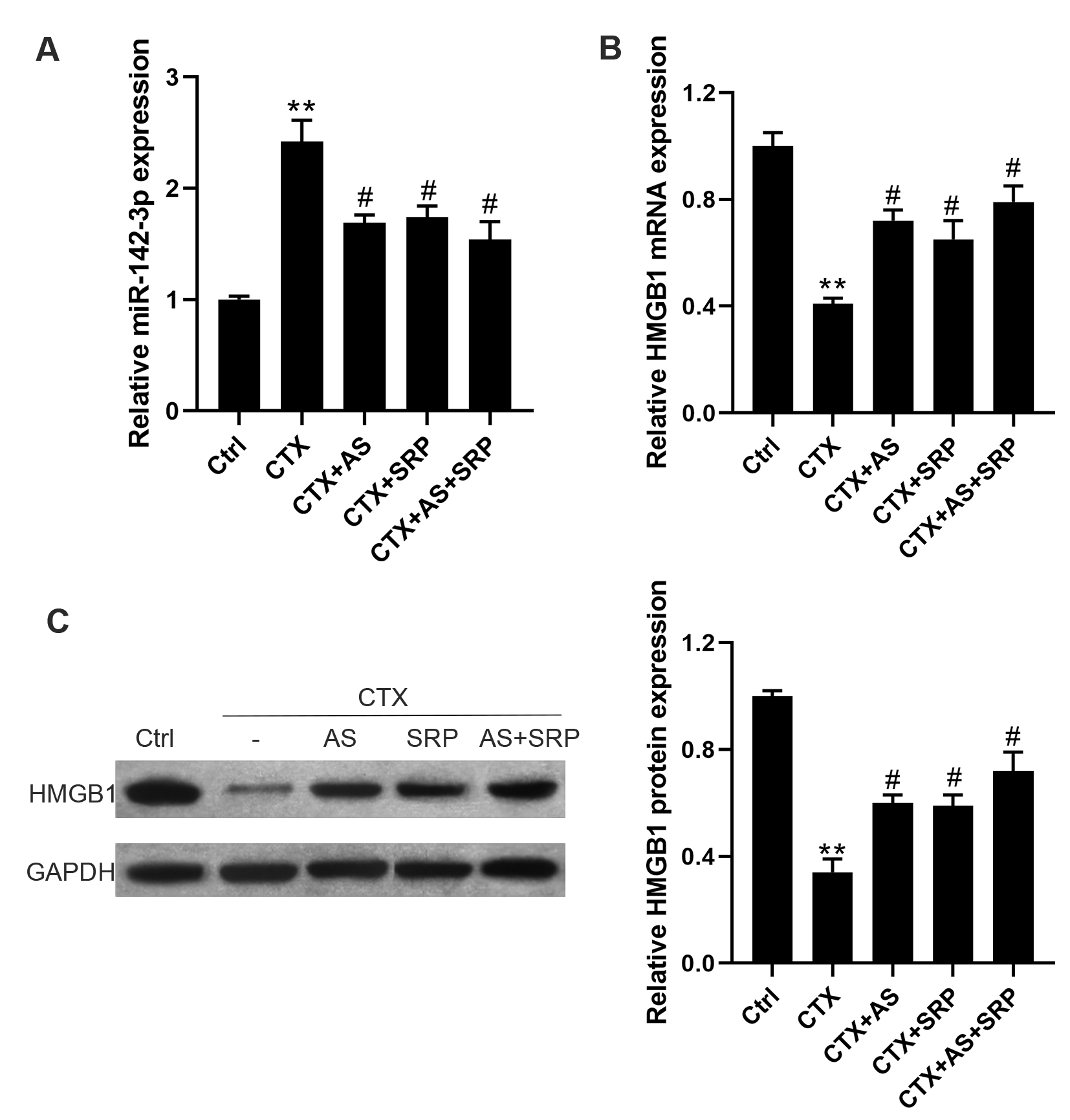


**Supplemental Figure 2. The expression of miR-142-3p and HMGB1 in BMHSCs isolated from mice in various groups. (A)** The expression level of miR-142-3p. **(B)** The mRNA expression level of HMGB1. **(C)** The protein expression level of HMGB1. Data were expressed as means±SD, ***P*<0.01 *vs.* Ctrl group; ^#^*P*<0.01 *vs.* CTX alone group; *n*=5.
